# Pancreatic Tuberculosis Abscess Successfully Treated With Serial Endoscopic Ultrasound-Guided Aspirations

**DOI:** 10.14309/crj.0000000000000291

**Published:** 2020-01-02

**Authors:** Hasan Maulahela, Ahmad Fauzi, Nur Rahadiani

**Affiliations:** 1 Division of Gastroenterology, Department of Internal Medicine, Cipto Mangunkusumo National General Hospital, Jakarta, Indonesia; 2 Department of Pathology Anatomy, Cipto Mangunkusumo National General Hospital, Jakarta, Indonesia

## CASE REPORT

**Figure 1.**
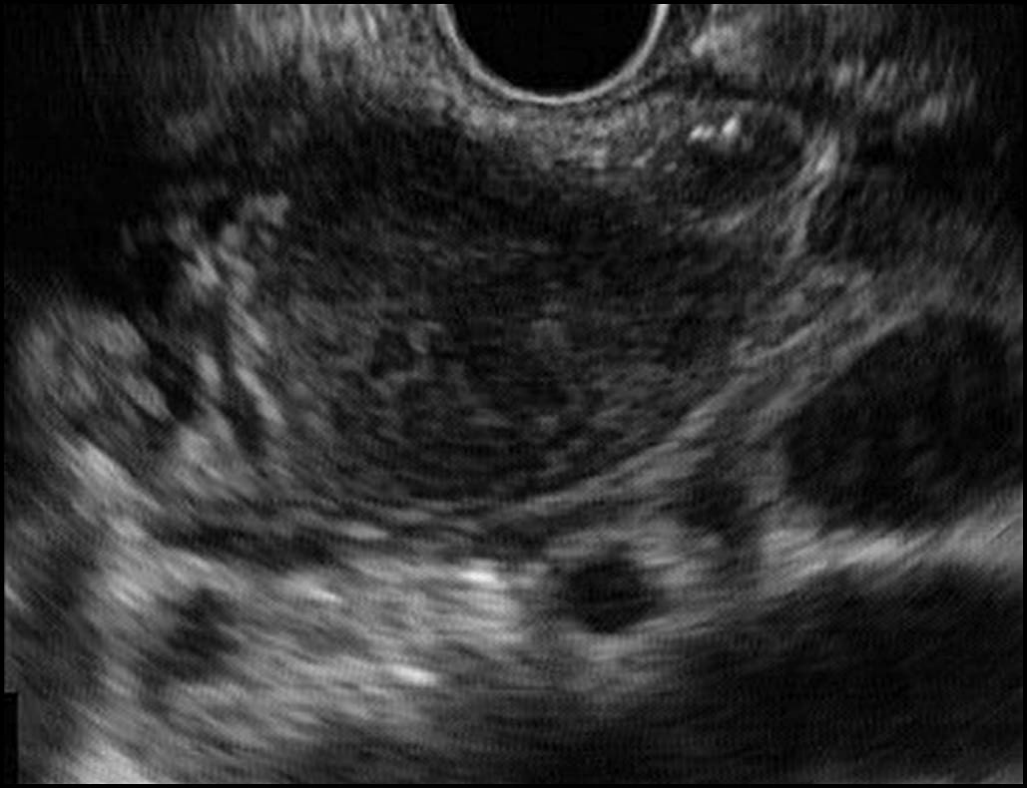
Hypoechoic lesion in the head of the pancreas.

**Figure 2.**
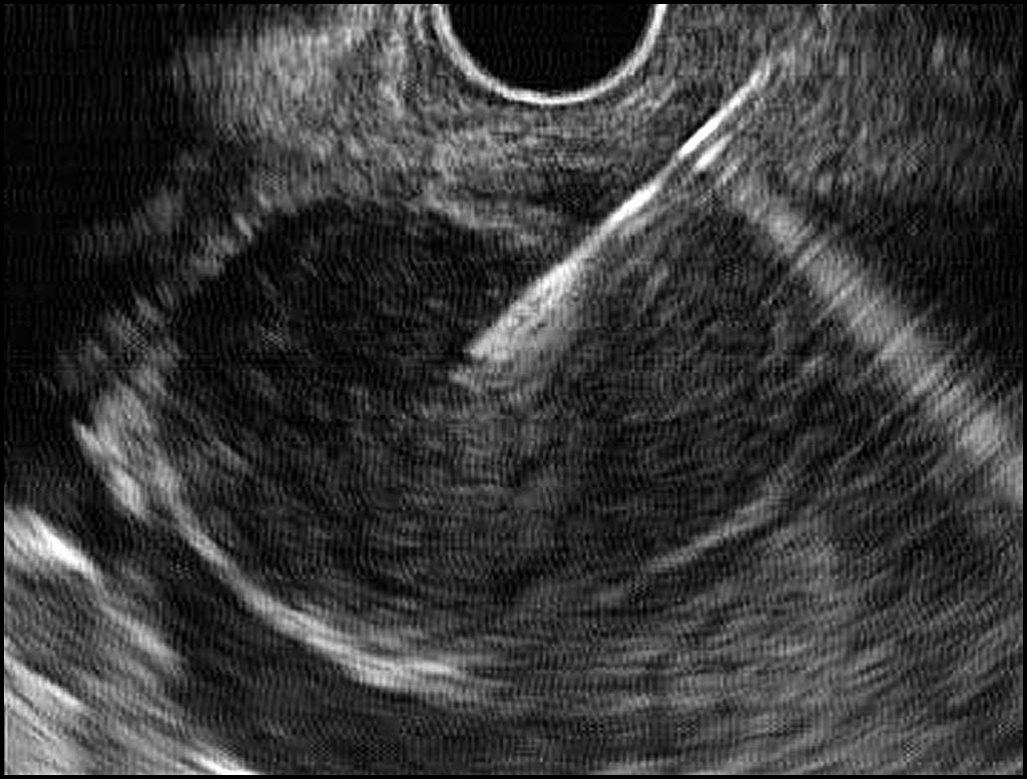
Fine-needle aspiration of the suspected lesion.

**Figure 3.**
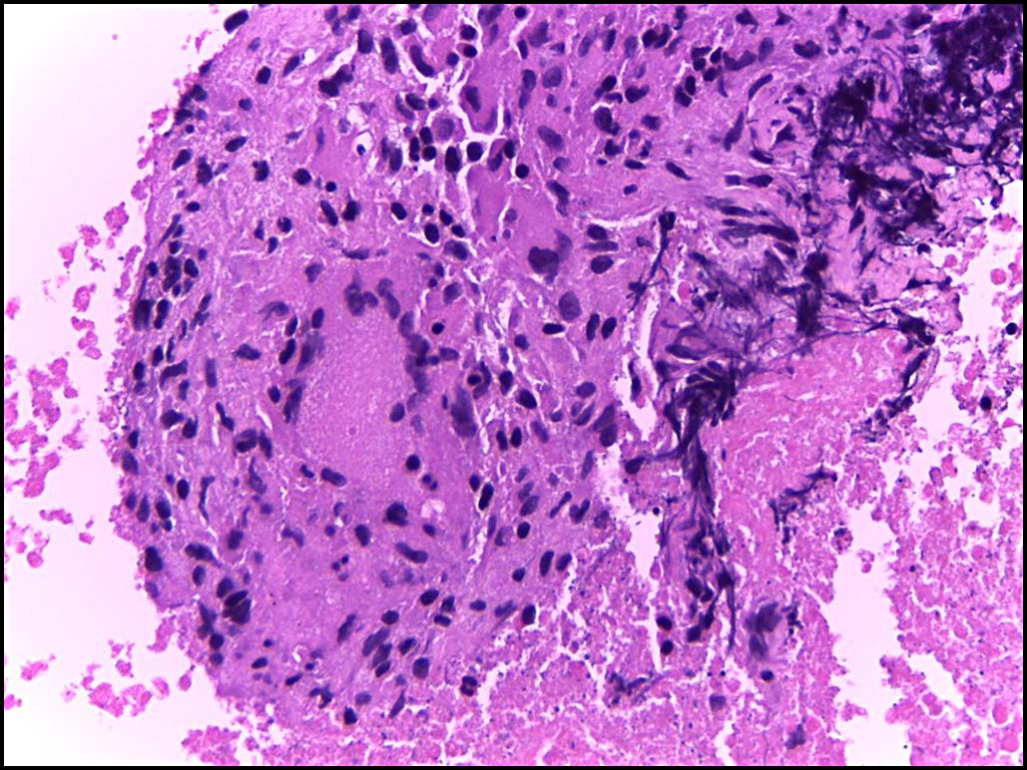
Cytology showing giant cell granuloma.

**Figure 4.**
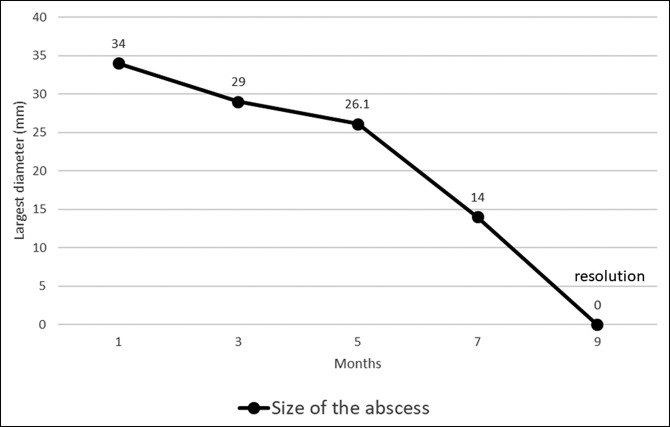
Graph showing abscess size reducing over time.

A 28-year-old woman suffered from abdominal pain for 3 weeks before admission. The patient also had weight loss and a decrease in appetite. From the physical examination, we found abdominal tenderness in the right upper quadrant. The result of magnetic resonance imaging was a suspected pancreatic cyst. Endoscopic ultrasound (EUS) showed a hypoechoic lesion with a definite border in the head of the pancreas with lymph node enlargement (Figure 1). We performed fine-needle aspiration of the lesion which oozed pus (Figure 2). The fluid was sent for cytology examination and tuberculosis polymerase chain reaction (PCR). The cytology result showed giant cell granuloma, and the tuberculosis PCR was positive (Figure 3). We performed aspiration of the abscess 3 times every 2 months until there was no pus aspirated and started administration of antituberculosis drugs for 9 months. EUS evaluation was performed every 2 months. After treatment with antituberculosis medication, the patient's abdominal pain was reduced, and she gained weight. EUS showed a decrease in size of the abscess and lymph nodes. The abscess resolved after 9 months (Figure 4).

Pancreatic tuberculosis is a rare form of extrapulmonary tuberculosis. Patients with pancreatic tuberculosis may have nonspecific symptoms such as abdominal pain, weight loss, weakness, or fever.^1^ On computed tomography imaging, pancreatic tuberculosis can mimic pancreatic carcinoma.^2^ The manifestation of pancreatic tuberculosis can present as an abscess or cystic mass. EUS is more sensitive in differentiating malignant and nonmalignant pancreatic lesions when compared with computed tomography.^3^ EUS-guided fine-needle aspiration allows us not only to obtain tissue samples for PCR diagnosis but also to perform therapeutic aspiration of the abscess.^4,5^ In this case, we treated the patient using standard triple therapy for 9 months, with a follow-up using EUS to observe the resolution of the pancreatic abscess.

## DISCLOSURES

Author contributions: H. Maulahela, A. Fauzi, and N. Rahadiani wrote the manuscript. H. Maulahela is the article guarantor.

Financial disclosure: None to report.

Informed consent was obtained for this case report.
